# Barriers to effective community health nursing: a quantitative analysis of challenges faced by nursing students in Saudi Arabia

**DOI:** 10.3389/fmed.2026.1870370

**Published:** 2026-08-19

**Authors:** Bandar Alhumaidi

**Affiliations:** Department of Community Health Nursing, College of Nursing, Taibah University, Medina, Saudi Arabia

**Keywords:** barriers to practice, clinical training, community health nursing, nursing education, nursing students, primary healthcare, Saudi Arabia, Vision 2030

## Abstract

**Introduction:**

Community health nursing (CHN) is central to primary healthcare delivery, but undergraduate nursing students may encounter educational, organizational, resource-related, and sociocultural barriers that affect their readiness for community-based practice. This study aimed to identify and quantify perceived barriers to CHN practice among nursing students in Saudi Arabia and to examine their relationships with self-reported educational preparedness and organizational support.

**Methods:**

A quantitative cross-sectional survey was conducted among nursing students enrolled in Saudi universities. A structured online questionnaire assessed perceived CHN practice barriers, educational preparedness, and organizational support. The instruments underwent cultural adaptation, forward-backward translation, expert-panel content validation, pilot testing, and internal-consistency assessment. Descriptive statistics, non-parametric tests, correlation analyses, and exploratory multiple linear regression were used.

**Results:**

A total of 139 nursing students completed the survey. Participants reported minor-to-moderate overall barriers (*M* = 2.69, SD = 0.96). Resource barriers were rated highest, with shortage of equipment (*M* = 2.93, SD = 1.38) and lack of educational materials (*M* = 2.86, SD = 1.32) identified as the most salient challenges. Educational preparedness (*M* = 3.25, SD = 0.98) and organizational support (*M* = 3.46, SD = 1.03) were rated moderately positive. Small-to-moderate positive correlations were observed between barriers and educational preparedness (*r* = 0.269, *p* = 0.001) and between barriers and organizational support (*r* = 0.242, *p* = 0.004); these findings were interpreted cautiously as potentially reflecting awareness effects, conceptual overlap, or shared-method variance. The multivariable regression model was not statistically significant and explained a small proportion of variance (*R*^2^ = 0.089, *p* = 0.052).

**Discussion:**

Nursing students in Saudi Arabia perceived meaningful, though not severe, barriers to effective CHN practice, with resource limitations and skills gaps as primary concerns. The findings support strengthened curriculum-practice integration, expanded simulation and community-immersion experiences, improved placement resources, and targeted faculty development aligned with healthcare transformation priorities.

## 1 Introduction

Community health nursing (CHN) represents a specialized domain of nursing practice focused on promoting and protecting the health of populations through assessment, policy development, and assurance of conditions enabling health (1). As healthcare systems worldwide transition toward community-based models of care delivery, the preparation of nursing students for effective practice in community settings has become increasingly critical (2). In Saudi Arabia, the national transformation agenda encapsulated in Vision 2030 has placed significant emphasis on strengthening primary healthcare infrastructure and expanding community-based health services, positioning CHN at the forefront of healthcare reform (3, 4).

The World Health Organization has consistently emphasized that well-prepared community health nurses are essential for achieving universal health coverage and improving population health outcomes (5). However, nursing students across various settings have reported substantial barriers to effective CHN practice during their clinical training (6, 7). These barriers span multiple domains, including knowledge and skills deficits, resource limitations, environmental and cultural challenges, curriculum inadequacies, and organizational constraints (8). Understanding the nature and magnitude of these barriers is essential for developing evidence-based strategies to strengthen nursing education and improve community health outcomes.

In the Saudi Arabian context, the healthcare system has undergone rapid transformation in recent decades. The establishment of the Health Sector Transformation Program under Vision 2030 has accelerated reforms in healthcare delivery, with particular emphasis on shifting from hospital-centered care to community-based prevention and health promotion (3, 9). This paradigm shift demands a nursing workforce well prepared to function in diverse community settings. However, the extent to which current nursing education programs adequately prepare students for this expanded role remains insufficiently examined (10, 11).

The Saudi undergraduate nursing curriculum is predominantly delivered as a four-year Bachelor of Science in Nursing (BSN) program, followed by a compulsory 12-month internship year in which students rotate through hospital and community settings under licensed supervision (10, 12). CHN-related content is typically introduced longitudinally across the curriculum: foundational concepts of primary healthcare, epidemiology, and health promotion are embedded in early theoretical courses (Years 1–2), while dedicated CHN courses—covering family health assessment, home visits, school health, occupational health, and communicable disease control—are concentrated in Years 3 and 4. Clinical placements in CHN generally occur during Year 3 or Year 4 and in the internship year, and most commonly take the form of rotations through Ministry of Health primary healthcare centers, school health units, and community-based organizations. Placement durations vary across institutions but typically range from 4 to 8 weeks of part-time exposure, which is considerably shorter than the hospital-based rotations in medical-surgical and maternal–child nursing (10, 11, 13, 14). Evidence from Saudi Arabia and the wider region suggests that this relatively limited exposure, combined with variable preceptor availability, gaps between theoretical content and field realities, and the historically low status of community practice as a career choice, may contribute to students’ uncertainty about their readiness for independent CHN practice (13–15). Situating the present study within this curricular context is essential for interpreting students’ perceptions of barriers, preparedness, and organizational support.

Several international studies have documented barriers to CHN practice among students, identifying common themes such as inadequate clinical exposure, resource constraints, and gaps between theoretical knowledge and practical application (16, 17). A recent systematic review of the theory–practice gap in nursing services highlighted that clinical life challenges, misconceptions about clinical nursing, and a lack of collaboration between universities and clinical settings were persistent barriers across multiple countries (15). Research in Middle Eastern contexts has additionally identified cultural sensitivity challenges, transportation limitations, and safety concerns as region-specific barriers (6, 18).

Despite this growing international literature, there is a notable paucity of research examining barriers to CHN practice specifically among Saudi nursing students. A recent study on CHN practices and learning needs in Saudi Arabia underscored the need for enhanced training and skill development among nurses working in primary healthcare centers (13). A multicenter study further found that community care is an undervalued career choice among Saudi nursing students, highlighting the need for career development plans that actively immerse students in community care activities (14). However, no comprehensive quantitative study has simultaneously examined barriers across multiple domains while also assessing their relationships with educational preparedness and organizational support in the Saudi context.

This study addresses this gap by employing a multi-dimensional quantitative approach to identify and quantify barriers to effective CHN practice among nursing students in Saudi Arabia. Specifically, the study aimed to: (1) assess perceived barriers to CHN practice across knowledge/skills, resource, and environmental/cultural domains; (2) evaluate students’ perceptions of educational preparedness and organizational support; (3) examine the relationships between perceived barriers, educational preparedness, and organizational support; and (4) determine whether selected demographic characteristics were associated with the perception of barriers. Given the exploratory nature of the work and the limited Saudi-specific evidence base, no directional hypotheses were formally pre-specified; however, in line with international literature (15–17), an inverse relationship between barriers and both educational preparedness and organizational support was tentatively expected.

## 2 Materials and methods

### 2.1 Study design

A quantitative, cross-sectional survey design was employed to assess barriers to effective CHN practice among nursing students in Saudi Arabia (19). The study is reported in accordance with the STROBE (Strengthening the Reporting of Observational Studies in Epidemiology) statement for cross-sectional research.

### 2.2 Setting and participants

The study targeted nursing students enrolled in accredited undergraduate nursing programs across Saudi Arabia. A convenience sampling strategy was used; participants were recruited from multiple universities through online platforms and institutional email distribution between December 2025 and January 2026. Inclusion criteria were: (a) current enrollment in an accredited BSN or equivalent undergraduate nursing program in Saudi Arabia, and (b) willingness to provide informed consent. The only exclusion criterion was the submission of an incomplete, duplicate, or invalid questionnaire with substantial missing data that precluded statistical analysis. Of the 156 survey responses initially received, 139 complete and valid responses were retained for analysis, yielding an effective completion rate of 89.1% among those who accessed the questionnaire.

### 2.3 Sample size

*A priori* sample-size estimation was performed using G*Power software (version 3.1) (20) based on the following parameters: medium effect size (*f*^2^ = 0.15), significance level (*α* = 0.05), statistical power (1 − *β* = 0.80), and six predictor variables for multiple linear regression, yielding a minimum required sample of 98 participants. The achieved sample of 139 complete responses exceeded this minimum requirement.

### 2.4 Instruments

The survey instrument comprised four sections.

#### 2.4.1 Section 1: demographic information

Eight items captured categorical participant characteristics including age group, gender, academic year (2nd, 3rd, 4th, or internship year, with internship year defined as the post-coursework supervised clinical year required for graduation), nursing program type (BSN, bridge program [defined as a diploma-to-BSN conversion pathway for practicing diploma-holders], or other), university, university region, CHN clinical rotation status, and duration of CHN clinical experience in weeks. Age and clinical-duration variables were intentionally collected in categories rather than as exact continuous values; therefore, these demographic variables are reported as frequencies and percentages and are not summarized as mean (SD).

#### 2.4.2 Section 2: barriers to practice scale

This 15-item scale was adapted, with written permission from the original author, from the BARRIERS to Research Utilization Scale developed by Funk et al. (21), a widely used instrument whose domain structure and item-response format have been applied across several nursing contexts. Items were reformulated to address perceived barriers to CHN practice rather than research utilization and organized into three conceptually grounded subscales: Knowledge and Skills Barriers (5 items), Resource Barriers (5 items), and Environmental and Cultural Barriers (5 items). Items were rated on a 5-point Likert scale ranging from 1 (Not a barrier) to 5 (Severe barrier).

#### 2.4.3 Section 3: educational preparedness assessment

This 12-item scale was developed by the author for the present study, drawing on constructs identified in the Saudi CHN literature (10, 11, 13, 14) and on the theory–practice-gap framework described by Singh et al. (15) and Vosoughi et al. (22). It assessed perceptions of educational preparation across three subscales: Curriculum Content (4 items), Clinical Training (4 items), and Self-Assessment of Preparedness (4 items). Items were rated on a 5-point Likert scale from 1 (Strongly Disagree) to 5 (Strongly Agree).

#### 2.4.4 Section 4: organizational support measure

This 10-item scale was also developed by the author for the present study, informed by the Organizational Support literature (8, 10, 22) and by Saudi Vision 2030 policy documents (3, 4, 9). It assessed organizational and policy support across three subscales: Institutional Support (4 items), Faculty Support (3 items), and Policy Environment (3 items), using the same 5-point Likert agreement format.

### 2.5 Adaptation, translation, and validation of instruments

Because the original BARRIERS Scale (21) and the newly developed Educational Preparedness and Organizational Support scales were in English, a structured adaptation, translation, and validation process was undertaken to ensure cultural and linguistic appropriateness for Saudi nursing students. The process comprised five sequential steps, following guidance derived from the WHO translation framework and from prior Arabic adaptations of nursing instruments (6, 18).

First, conceptual adaptation of the BARRIERS Scale was performed by the author in consultation with two senior CHN faculty members; items referring to research utilization were rephrased to refer to CHN practice in community and primary care settings, while preserving the original domain structure. The Educational Preparedness Assessment and Organizational Support Measure were generated *de novo* for this study based on an item pool derived from the literature (10, 11, 13–15, 22) and from Vision 2030 policy documents (3, 4).

Second, forward translation of all instruments from English to Modern Standard Arabic was independently carried out by two bilingual nursing academics whose first language is Arabic. Discrepancies were resolved through consensus in the presence of the author to produce a reconciled Arabic version.

Third, backward translation of the reconciled Arabic version into English was performed by a third bilingual translator (a nursing faculty member not involved in the forward translation and blinded to the original wording). The back-translated version was then compared with the original English instruments; minor semantic discrepancies were resolved by the author in consultation with the translators, and no items required substantive rewording.

Fourth, content validity was evaluated by an expert panel of five faculty members (three CHN specialists, one educational assessment specialist, and one biostatistician affiliated with Saudi universities). Experts rated each item for relevance, clarity, and cultural appropriateness on a 4-point scale; items with an item-level content validity index (I-CVI) below 0.78 were revised. All retained items achieved I-CVI values ≥ 0.80, and the scale-level content validity index (S-CVI/Ave) exceeded 0.90 for each of the three instruments, supporting acceptable content validity.

Fifth, the Arabic questionnaire was pilot-tested with 15 nursing students (who were not included in the main analysis) to assess clarity, average completion time, and technical functioning of the online platform. Pilot feedback led to minor formatting revisions but no changes to item content. Internal consistency of the instruments in the main sample was assessed using Cronbach’s alpha (23) (Table 1).

**Table 1:** Internal consistency reliability of study instruments.

Scale	Subscale	Items	α
Barriers to practice	Knowledge and Skills	5	0.893
	Resource Barriers	5	0.875
	Environmental and Cultural	5	0.889
	Total	15	0.945
Educational preparedness	Curriculum Content	4	0.916
	Clinical Training	4	0.893
	Self-Assessment	4	0.878
	Total	12	0.947
Organizational support	Institutional Support	4	0.921
	Faculty Support	3	0.915
	Policy Environment	3	0.949
	Total	10	0.965

α, Cronbach’s alpha coefficient.

This table shows how consistently the questionnaire measured each concept. Cronbach’s alpha is a single value between 0 and 1 indicating whether students answered the questions belonging to the same scale in a similar way. Values of 0.70 or above are generally regarded as acceptable, and higher values indicate greater consistency. Every scale and subscale in this study fell between 0.875 and 0.965, meaning that the questions within each scale held together well and can reasonably be combined into one score.

### 2.6 Data collection

Data were collected through a structured online questionnaire administered via Google Forms. The survey link was distributed through university learning management systems and student email lists. Participants were provided with a digital informed consent form prior to accessing the survey. The questionnaire took approximately 10–15 min to complete. Data collection occurred over a four-week period from late December 2025 to January 2026.

### 2.7 Ethical considerations

Ethical approval was obtained from the Research Ethics Committee of the College of Nursing, Taibah University (IRB: TUCN-REC 3-122025, approved 23 December 2025). Written permission to adapt the BARRIERS Scale was obtained from the original instrument developer. Informed consent was obtained electronically from all participants; participation was voluntary and anonymous, with no personal identifiers collected.

### 2.8 Data analysis

Data were analyzed using Python (version 3.12) with the SciPy, NumPy, and Pandas libraries. Descriptive statistics (means, standard deviations, medians, and ranges) were calculated for scale and subscale scores; frequencies and percentages were computed for categorical demographic variables. Because age and CHN clinical duration were collected as categories rather than exact continuous values, they were not summarized using mean (SD). Normality of the three composite scale scores was examined using the Shapiro–Wilk test (24) (Barriers: *W* = 0.972, *p* = 0.008; Educational Preparedness: *W* = 0.947, *p* < 0.001; Organizational Support: *W* = 0.943, *p* < 0.001), indicating deviation from normality. Accordingly, non-parametric tests were used for group comparisons of ordinal Likert-based composite scores: the Mann–Whitney *U* test for two-group comparisons and the Kruskal–Wallis H test for multi-group comparisons (25). Both Pearson and Spearman correlation coefficients were computed; Pearson coefficients are reported in the main text because all composite scores were treated as approximately interval-level summed Likert scales with similar shape, while Spearman coefficients were inspected as a sensitivity check and yielded substantively identical conclusions. Multiple linear regression was used in an exploratory manner to examine predictive relationships; model assumptions (linearity, homoscedasticity, independence, and multicollinearity) were inspected using residual plots and variance inflation factors (VIFs < 5 for all predictors). A significance level of *p* < 0.05 (two-tailed) was adopted throughout.

## 3 Results

### 3.1 Demographic characteristics

A total of 139 nursing students completed the survey. Because age and clinical duration were captured as categorical variables, these demographic variables are summarized using frequencies and percentages rather than mean (SD). The majority were female (*n* = 134, 96.4%), aged 21–23 years (*n* = 72, 51.8%), and enrolled in BSN programs (*n* = 132, 95.0%). Fourth-year students comprised the largest academic group (*n* = 54, 38.8%), followed by second-year (*n* = 46, 33.1%) and third-year students (*n* = 32, 23.0%). The majority of respondents were from the Western Region (*n* = 120, 86.3%). More than half of the participants had not yet started their CHN clinical rotation (*n* = 81, 58.3%), while 28.8% (*n* = 40) were currently enrolled and 12.9% (*n* = 18) had completed it. Complete demographic data are presented in Table 2.

**Table 2:** Demographic characteristics of participants (*N* = 139).

Variable	Category	*n*	%
Age group	18–20 years	62	44.6
	21–23 years	72	51.8
	24–26 years	2	1.4
	≥27 years	3	2.2
Gender	Female	134	96.4
	Male	5	3.6
Academic year	2nd Year	46	33.1
	3rd Year	32	23.0
	4th Year	54	38.8
	Internship Year	7	5.0
Program type	BSN	132	95.0
	Bridge Program	1	0.7
	Other	6	4.3
University region	Western Region	120	86.3
	Eastern Region	9	6.5
	Southern Region	4	2.9
	Northern Region	4	2.9
	Central Region	2	1.4
Clinical rotation	Completed	18	12.9
	Currently enrolled	40	28.8
	Not yet started	81	58.3
Clinical duration	Not applicable	83	62.4
	<4 weeks	24	18.0
	4–8 weeks	18	13.5
	>8 weeks	8	6.0

This table describes who took part in the study. Each row is one background characteristic, such as age or academic year. The column headed n gives the number of students in each category, and the column headed % gives the share of the whole sample that this number represents. Read together, the rows show that the participants were almost all female undergraduate BSN students, mainly in their second to fourth year, studying largely in the Western Region, and that most had not yet begun their community health nursing clinical rotation.

### 3.2 Instrument reliability

Internal consistency analysis demonstrated high reliability across all scales and subscales, with Cronbach’s alpha coefficients ranging from 0.875 (Resource Barriers) to 0.965 (Total Organizational Support). All values substantially exceeded the accepted threshold of 0.70, indicating strong internal consistency (23) (Table 1).

### 3.3 Perceived barriers to community health nursing practice

Participants reported minor-to-moderate overall barriers (*M* = 2.69, SD = 0.96). Among the three subscales, Resource Barriers received the highest mean (*M* = 2.72, SD = 1.03), followed closely by Knowledge and Skills Barriers (*M* = 2.71, SD = 1.07) and Environmental and Cultural Barriers (*M* = 2.65, SD = 1.08). All subscale means fell below the midpoint of the 5-point scale, suggesting that barriers were generally perceived at minor-to-moderate severity rather than as major obstacles (Table 3).

**Table 3:** Descriptive statistics for study subscales (*N* = 139).

Subscale	*M*	SD	Mdn	Range
Knowledge and skills barriers	2.71	1.07	2.80	1.00–5.00
Resource barriers	2.72	1.03	3.00	1.00–5.00
Environmental and Cultural barriers	2.65	1.08	2.80	1.00–5.00
Overall barriers to practice	2.69	0.96	2.87	1.00–5.00
Curriculum content	3.28	1.13	3.00	1.00–5.00
Clinical training	3.32	1.02	3.25	1.00–5.00
Self-assessment of preparedness	3.16	1.08	3.00	1.00–5.00
Overall educational preparedness	3.25	0.98	3.17	1.00–5.00
Institutional support	3.39	1.04	3.25	1.00–5.00
Faculty support	3.43	1.08	3.33	1.00–5.00
Policy environment	3.59	1.17	3.67	1.00–5.00
Overall organizational support	3.46	1.03	3.30	1.00–5.00

M, mean; SD, standard deviation; Mdn, median. Barriers rated 1–5 (not a barrier to severe barrier); preparedness and support rated 1–5 (strongly disagree to strongly agree).

This table summarizes how students answered each group of questions on the 1–5 rating scales. The mean (M) is the average rating: for the barrier subscales a higher average means the barrier was felt to be more severe, whereas for the preparedness and support subscales a higher average means stronger agreement. The standard deviation (SD) shows how far apart students’ answers were, so a larger SD means students differed more from one another. The median (Mdn) is the middle answer once all responses are placed in order, and the range shows the lowest and highest ratings given. In practical terms, every barrier average fell below the scale midpoint of 3, indicating barriers of minor to moderate severity, while the preparedness and support averages sat above 3, indicating moderate agreement.

### 3.4 Item-level analysis of barriers

At the item level, the five highest-rated barriers were: shortage of equipment (*M* = 2.93, SD = 1.38), lack of educational materials (*M* = 2.86, SD = 1.32), inadequate communication skills (*M* = 2.81, SD = 1.34), lack of practical assessment skills (*M* = 2.78, SD = 1.35), and limited home-visit competence (*M* = 2.75, SD = 1.17). The two highest-rated items both belonged to the Resource Barriers subscale, underscoring resource constraints as primary obstacles (13). The complete ranking is presented in Table 4.

**Table 4:** Barrier items ranked by mean score (*N* = 139).

Rank	Item	Domain	*M*	SD	% 4–5
1	Shortage of equipment for CHN services	Resource	2.93	1.38	33.8
2	Lack of educational materials and resources	Resource	2.86	1.32	35.3
3	Inadequate communication skills	Knowledge	2.81	1.34	29.5
4	Lack of practical skills in community assessment	Knowledge	2.78	1.35	31.7
5	Limited competence in home-visit procedures	Knowledge	2.75	1.17	25.9
6	Geographic accessibility issues	Environ.	2.70	1.23	25.9
7	Community resistance to health services	Environ.	2.68	1.38	27.3
8	Cultural sensitivity challenges	Environ.	2.66	1.32	25.9
9	Inadequate clinical supervision	Resource	2.65	1.25	23.0
10	Insufficient knowledge about CHN	Knowledge	2.64	1.21	23.0
11	Language barriers	Environ.	2.61	1.31	23.7
12	Safety concerns in community	Environ.	2.60	1.27	22.3
13	Difficulty in health-education techniques	Knowledge	2.59	1.33	23.7
14	Insufficient transportation	Resource	2.59	1.18	21.6
15	Limited time for community activities	Resource	2.58	1.14	20.1

% 4–5, percentage of respondents rating item as Major or Severe barrier.

This table lists the 15 individual barrier statements in order, from the one students rated most severe to the one they rated least severe. *M* is the average rating on the 1–5 scale, SD shows how much students differed from one another in rating that statement, and the final column gives the percentage of students who judged the item to be a major or severe barrier (a rating of 4 or 5). The two statements at the top of the list, shortage of equipment and lack of educational materials, both concern resources, and about one third of students regarded each of them as a major or severe obstacle.

The highest proportions of students rating items as major or severe barriers (scores 4–5) were observed for: lack of educational materials (35.3%), shortage of equipment (33.8%), lack of practical skills (31.7%), and inadequate communication skills (29.5%). These percentages indicate that approximately one-third of students considered resource and skills deficits to be substantial barriers.

### 3.5 Educational preparedness

Students reported moderate agreement with educational preparedness statements (*M* = 3.25, SD = 0.98). Clinical Training received the highest rating (*M* = 3.32, SD = 1.02), followed by Curriculum Content (*M* = 3.28, SD = 1.13) and Self-Assessment (*M* = 3.16, SD = 1.08). Clinical instructor availability was rated highest at the item level (*M* = 3.45, SD = 1.15), whereas readiness for independent community work was rated lowest (*M* = 3.12, SD = 1.24). These findings suggest some uncertainty about readiness for independent community practice (15, 16).

### 3.6 Organizational support

Organizational support was rated moderately positive overall (*M* = 3.46, SD = 1.03). Policy Environment received the highest subscale rating (*M* = 3.59, SD = 1.17), followed by Faculty Support (*M* = 3.43, SD = 1.08) and Institutional Support (*M* = 3.39, SD = 1.04). The item on Vision 2030’s positive impact on nursing education received the highest individual score (*M* = 3.70, SD = 1.21), indicating student awareness of the national policy framework (3, 4).

### 3.7 Barriers by demographic characteristics

Non-parametric analyses revealed no statistically significant differences in overall barrier scores across demographic groups. Detailed demographic comparison results are provided in Supplementary Table S1. The Mann–Whitney *U* test showed no significant gender difference (*U* = 298.5, *p* = 0.684), although this comparison was markedly underpowered because of the small number of male respondents (*n* = 5). Kruskal–Wallis tests indicated no significant differences across academic years (*H* = 1.70, *p* = 0.637), clinical rotation status (*H* = 1.77, *p* = 0.412), or university region (*H* = 1.43, *p* = 0.233). While internship-year students reported numerically lower barrier scores (*M* = 2.30, SD = 1.16), the very small subgroup size (*n* = 7) precludes substantive interpretation.

### 3.8 Correlations among study variables

Pearson correlation analysis revealed small-to-moderate positive correlations between overall barriers and educational preparedness (*r* = 0.269, *p* = 0.001) and between overall barriers and organizational support (*r* = 0.242, *p* = 0.004). The strongest correlation was observed between educational preparedness and organizational support (*r* = 0.825, *p* < 0.001). At the subscale level, Knowledge and Skills Barriers correlated most strongly with Curriculum Content (*r* = 0.318, *p* < 0.001). Spearman coefficients yielded substantively identical results and are therefore not reported separately.

### 3.9 Multiple regression analysis

An exploratory multiple linear regression examined educational preparedness and organizational support subscales as predictors of overall barrier perceptions. The overall model approached, but did not reach, conventional statistical significance [*F*(6, 132) = 2.15, *p* = 0.052] and explained a small proportion of variance (*R*^2^ = 0.089, Adjusted *R*^2^ = 0.048). Curriculum Content (*β* = 0.204, *p* = 0.166) and Faculty Support (*β* = 0.209, *p* = 0.238) showed the largest standardized coefficients, but none of the individual predictors reached statistical significance. Given that the model was not significant, these coefficients should not be interpreted substantively. The modest overall predictive power suggests that perceived barriers are multifactorial and likely influenced by variables beyond the educational and organizational constructs measured here (Table 5).

**Table 5:** Multiple regression: exploratory predictors of barrier perceptions.

Predictor	*B*	SE	*β*	*t*	*p*
(Constant)	1.821	0.295	—	6.18	<0.001
Curriculum content	0.174	0.125	0.204	1.39	0.166
Clinical training	0.005	0.147	0.005	0.03	0.975
Self-assessment	0.054	0.124	0.061	0.44	0.663
Institutional support	−0.137	0.174	−0.148	−0.79	0.434
Faculty support	0.186	0.157	0.209	1.18	0.238
Policy environment	−0.017	0.143	−0.020	−0.12	0.907

B, unstandardized coefficient; SE, standard error; β, standardized coefficient. *R*^2^ = 0.089, Adjusted *R*^2^ = 0.048, *F*(6, 132) = 2.15, *p* = 0.052. The overall model did not reach statistical significance and results are reported for exploratory purposes.

This table tests whether students’ ratings of educational preparedness and organizational support can be used to predict how severe they perceived barriers to be. Each row represents one possible predictor. B indicates how much the barrier score would be expected to change for each one-point change in that predictor, SE indicates how precise that estimate is, and *β* places all predictors on a common scale so that their relative strength can be compared. The p value indicates how likely a result of this size could have arisen by chance, with values below 0.05 conventionally treated as statistically significant. No predictor reached this threshold, and the model as a whole did not either, accounting for less than 9% of the differences between students. The table is therefore presented as an exploratory result only and indicates that perceived barriers are shaped by factors beyond those measured in this study.

## 4 Discussion

This study provides a quantitative assessment of barriers to effective CHN practice among nursing students in Saudi Arabia. The findings depict a nuanced picture in which students perceive minor-to-moderate barriers across multiple domains, with resource limitations and skills deficits emerging as the most prominent concerns.

### 4.1 Barriers to community health nursing practice

The overall minor-to-moderate level of perceived barriers (*M* = 2.69) suggests that while students do not view CHN as overwhelmingly obstructed, meaningful challenges warrant attention. The finding that resource barriers ranked highest is consistent with international literature highlighting equipment and material shortages as persistent obstacles (6, 13). Alruwaili et al. (26), in their systematic review of technology and innovation integration in CHN practice in Saudi Arabia, similarly identified infrastructure deficiencies as a recurring challenge. In the context of Vision 2030’s expansion of community-based services (3, 4), this finding underscores the need for adequate resource investment.

The prominence of knowledge and skills barriers, particularly communication skills (ranked 3rd) and practical assessment skills (ranked 4th), aligns with multiple studies documenting the theory–practice gap in nursing education (15–17). Ugwu et al. (17) reported that nursing students frequently experience clinical life challenges and misconceptions that hinder translation of theoretical knowledge into practice. This skills deficit may be partly attributable to limited CHN clinical exposure, as 58.3% of participants had not yet begun their CHN rotation.

Environmental and cultural barriers, including geographic accessibility (*M* = 2.70) and community resistance (*M* = 2.68), reflect Saudi Arabia’s distinctive sociocultural and geographic landscape (6, 18). Almegewly et al. (6) found that Saudi nursing students perceived cultural barriers as significant impediments to home-healthcare practice, consistent with the present findings. The relatively lower ranking of language barriers (*M* = 2.61) may reflect the predominantly Arabic-speaking study population.

### 4.2 Educational preparedness

The moderate educational preparedness scores (*M* = 3.25) indicate a generally positive but not fully satisfactory perception of preparation. The pattern of high clinical-instructor availability ratings (*M* = 3.45) alongside lower independence confidence (*M* = 3.12) reveals a pedagogical tension: students appreciate faculty support but remain uncertain about autonomous practice (16). This mirrors findings from Singh et al. (15), whose systematic review identified the need for culture-based content and clinically grounded curricula to bridge the theory–practice gap.

Strategies such as simulation-based learning (27), community-immersion experiences, and problem-based learning may help address this gap. Alrashidi et al. (28) demonstrated in their systematic review that simulation effectively improves nursing students’ self-confidence in clinical practice, supporting the case for expanded simulation integration in CHN curricula.

### 4.3 Organizational support and policy environment

The relatively positive organizational support perception (*M* = 3.46) and notably high Vision 2030 impact rating (*M* = 3.70) are encouraging findings. Al Khashan et al. (9) documented that Vision 2030 primary healthcare reforms have created new opportunities for community-based nursing practice. The present findings suggest that students perceive these policy-level changes positively (3, 4). However, the comparatively lower institutional support subscale rating (*M* = 3.39) indicates incomplete translation of policy aspirations into institutional resources, representing a critical intervention area (10, 11).

### 4.4 Interpreting the counterintuitive positive associations

The small-to-moderate positive correlations between perceived barriers and both educational preparedness (*r* = 0.269) and organizational support (*r* = 0.242) are counterintuitive and warrant cautious interpretation. On the basis of international literature (15–17), an inverse association had been tentatively expected. Several non-mutually exclusive explanations should be considered. First, an awareness effect is plausible: students with stronger preparation and greater engagement with faculty may be better equipped to recognize gaps between ideal and actual CHN practice, and may therefore rate barriers higher rather than lower (21, 22). Second, because 58.3% of respondents had not yet begun their CHN rotation, many ratings may reflect anticipated rather than directly experienced barriers, which are likely to be more sensitive to conceptual exposure in coursework than to practical realities. Third, shared-method variance is a real possibility, given that all three constructs were self-reported on similar Likert formats within the same online instrument; this can inflate inter-scale correlations, particularly between closely related constructs (as reflected by the very high preparedness–support correlation, *r* = 0.825). Fourth, conceptual overlap between items—for example, preparedness items that prompt reflection on curricular weaknesses—may prime barrier recognition. These findings should therefore not be read as evidence that preparation or organizational support increases barriers, but rather as a signal that barrier perceptions in this population are driven by factors not fully captured by educational and organizational constructs alone (7, 8).

Consistent with this interpretation, the exploratory regression model was not statistically significant (*p* = 0.052) and explained only a small proportion of variance (*R*^2^ = 0.089). No individual predictor reached significance, precluding substantive claims about directional effects. Future studies should include variables such as clinical-experience dose, preceptor quality, prior community exposure, and individual dispositional factors (e.g., clinical self-efficacy), which may better account for variation in perceived barriers (7, 8, 22, 29).

### 4.5 Demographic patterns

The absence of significant demographic differences suggests that barriers are systemic rather than group-specific, implying that interventions should target system-level change (10, 12). The numerical trend toward lower barrier scores among internship students (*M* = 2.30), while not statistically significant, warrants further investigation with larger internship-stage samples and may suggest that accumulated experience helps mitigate perceived barriers over time (29).

### 4.6 Implications for nursing curricula and practice

The findings support several concrete recommendations for Saudi BSN programs and CHN faculty. At the curriculum level, the high prominence of communication skills and community-assessment skills as barriers suggests that CHN curricula should strengthen structured, competency-based teaching of therapeutic communication, motivational interviewing, and family- and community-level assessment beginning no later than Year 2, rather than concentrating these skills in Year 4. Vertical integration of CHN concepts—with brief community-exposure modules embedded in early foundational courses and culminating in a longitudinal community-based clinical experience in Year 4 and the internship year—is likely to produce more graduated, experience-based preparation than the current short block rotations.

At the clinical-training level, CHN placements should be lengthened where feasible (ideally ≥ 8 weeks of full-time or equivalent part-time exposure) and diversified beyond primary healthcare centers to include home-visit rotations, school health units, occupational health settings, and chronic-disease follow-up programs. Given the low confidence for independent community work observed in the sample, each placement should include explicit progression from observation, to co-practice with a preceptor, to graduated independent practice under supervision.

At the pedagogical level, simulation-based learning should be integrated as a mandatory component of CHN courses. Scenarios prioritized for simulation—informed by the item-level ranking of barriers in this study—should include home-visit procedures, cross-cultural health education, family interviewing, and management of community-level resistance to health services (27, 28). Standardized community members and low-fidelity role-play can be used where high-fidelity infrastructure is unavailable. Faculty development should accompany this expansion, given that faculty support emerged as an important correlate of preparedness in this sample.

At the resource and institutional level, the prominence of shortage of equipment and lack of educational materials as the two highest-rated barriers points to a clear, actionable priority: universities and Ministry of Health affiliates should ensure that CHN placement sites are equipped with basic assessment tools, health-education materials in Arabic, and reliable transportation for home visits (13, 26). Formal university–primary-healthcare-center partnerships with memoranda of understanding specifying student roles, preceptor responsibilities, and resource provision would address several of these barriers simultaneously.

At the policy level, the positive perception of Vision 2030 provides a favorable environment for these reforms (3, 4, 9). Aligning CHN curriculum outcomes explicitly with Vision 2030 Health Sector Transformation Program objectives, and positioning community practice as a valued career pathway, may further reduce the barriers identified in this and in prior Saudi studies (13, 14).

### 4.7 Strengths and limitations

This study has several strengths. It addresses an underexplored area of Saudi nursing education using a multidimensional quantitative approach, includes both scale-level and item-level analyses, reports an *a priori* sample-size calculation, follows STROBE reporting principles, and describes a structured process of instrument translation, content validation, pilot testing, and reliability assessment. The item-level results also provide actionable evidence for curriculum planning, clinical placement improvement, and institutional resource allocation.

However, several important limitations should be considered when interpreting these findings. First, the cross-sectional design precludes causal inferences. Second, the convenience sampling strategy produced a sample that was heavily skewed toward the Western Region (86.3%) and toward female BSN students (96.4%), and the very small male subgroup (*n* = 5) meant that gender comparisons were severely underpowered; overall generalizability to male students and to other regions of Saudi Arabia is therefore limited. Third, a response rate at the institutional level could not be calculated because the survey was distributed via open links and mailing lists whose total reach could not be precisely quantified; this precludes formal assessment of non-response bias. Fourth, a majority of participants (58.3%) had not yet started their CHN clinical rotation, meaning that many barrier ratings likely reflect anticipated or perceived barriers rather than barriers directly encountered in practice. Fifth, although the adaptation and validation process followed established steps, the Educational Preparedness and Organizational Support scales were newly developed for this study and have not yet been subjected to confirmatory factor analysis or criterion-related validity testing in an independent Saudi sample. Sixth, self-reported data collected through a single online instrument are subject to response and shared-method biases. Seventh, the exploratory regression model was not statistically significant and explained only a small proportion of variance, and relevant covariates such as clinical preceptorship quality, prior community exposure, and clinical self-efficacy were not measured. Finally, the absence of a qualitative component limits the depth of understanding of the mechanisms underlying students’ perceptions. Future research should employ mixed-methods designs with larger, nationally representative, and more gender-diverse samples, ideally sampling students at defined stages of CHN exposure (19).

## 5 Conclusion

This study provides an initial quantitative assessment of perceived barriers to effective CHN practice among nursing students in Saudi Arabia. Students perceived minor-to-moderate barriers across knowledge/skills, resource, and environmental/cultural domains, with resource constraints and skills deficits as the most prominent concerns. The positive perception of organizational support, particularly regarding Vision 2030 initiatives, provides a favorable foundation for targeted improvement. However, the counterintuitive positive associations between barriers and both preparedness and organizational support, together with the non-significant regression model, indicate that the drivers of barrier perceptions in this population extend beyond the educational and organizational constructs measured here and should be investigated in adequately designed follow-up studies. Given the sampling and clinical-exposure limitations noted above, the findings are best regarded as hypothesis-generating rather than definitive. Nevertheless, they converge with prior Saudi and international evidence in pointing toward concrete, actionable priorities for CHN curriculum reform, simulation integration, expanded community placements, and resource investment as Saudi Arabia continues its healthcare transformation under Vision 2030.

## Data Availability

The raw data supporting the conclusions of this article will be made available by the authors, without undue reservation.

## Supplementary material

Supplementary Table S1 Non-parametric comparisons of overall barrier scores across demographic groups (*N* = 139).
