# Supplementary material for: Barriers to effective community health nursing: a quantitative analysis of challenges faced by nursing students in Saudi Arabia
Source: Front Med (Lausanne). 2026 Aug 19;13:1870370. doi: 10.3389/fmed.2026.1870370 (PMC13533645; doi:10.3389/fmed.2026.1870370)
Supplement: Supplementary file 1 [file Table_1.docx]

# Supplementary Material

**Supplementary Table S1. Comparison of mean barrier scores by demographics.**

| **Variable** | **n** | **M** | **SD** | **Statistic** | **p** |
| --- | --- | --- | --- | --- | --- |
| Gender |  |  |  | U = 298.5 | 0.684 |
| Female | 134 | 2.69 | 0.96 |  |  |
| Male | 5 | 2.73 | 1.11 |  |  |
| Academic year |  |  |  | H = 1.70 | 0.637 |
| 2nd Year | 46 | 2.72 | 1.09 |  |  |
| 3rd Year | 32 | 2.81 | 0.99 |  |  |
| 4th Year | 54 | 2.66 | 0.81 |  |  |
| Internship | 7 | 2.30 | 1.16 |  |  |
| Clinical rotation |  |  |  | H = 1.77 | 0.412 |
| Completed | 18 | 2.59 | 1.05 |  |  |
| Enrolled | 40 | 2.58 | 0.95 |  |  |
| Not started | 81 | 2.78 | 0.96 |  |  |

U, Mann-Whitney U; H, Kruskal-Wallis H. Significance level: p < 0.05.
